# Stress-induced ribosomal heterogeneity in *Saccharomyces cerevisiae*: from protein paralogs to regulatory noncoding RNAs

**DOI:** 10.1093/femsyr/foaf050

**Published:** 2025-09-11

**Authors:** Agata Tyczewska, Kamilla Bąkowska-Żywicka

**Affiliations:** Institute of Bioorganic Chemistry Polish Academy of Sciences, Noskowskiego St. 12/14, 61-704 Poznań, Poland; Institute of Bioorganic Chemistry Polish Academy of Sciences, Noskowskiego St. 12/14, 61-704 Poznań, Poland

**Keywords:** *Saccharomyces cerevisiae*, ribosome, ribosome heterogeneity, specialized ribosomes, ribosome-associated noncoding RNAs, abiotic stress

## Abstract

Ribosomes, once considered uniform protein biosynthesis machines, are now recognized as heterogeneous and dynamic entities with specialized functions. In *Saccharomyces cerevisiae*, ribosomal heterogeneity arises from variability in ribosomal protein (RP) composition, rRNA sequence polymorphisms, post-transcriptional modifications, and associations with ribosome-associated factors and noncoding RNAs. RP gene (RPG) paralogs and their differential expression influence growth, stress resistance, and drug responses. Introns and untranslated regions in RPGs regulate expression under stress, while ribosome composition adjusts to environmental cues *via* altered RP stoichiometry and post-translational modifications, such as phosphorylation and ubiquitination. Additionally, ribosome-associated factors contribute to selective translation of specific mRNA subsets. Ribosomal RNA heterogeneity, though less studied in yeast, is evident through polymorphisms in rDNA arrays and post-transcriptional modifications like pseudouridylation and 2′-O-ribose methylation. Furthermore, transient associations with small noncoding RNAs (e.g. tRNA-, snoRNA-, and mRNA-derived fragments) modulate translation in a stress-dependent manner, supporting the concept of specialized ribosomes. Despite growing evidence, functional significance of ribosome specialization remains under debate. Future research aims to uncover the extent, regulation, and biological roles of ribosome heterogeneity across organisms and conditions. Emerging tools such as ribosome sequencing, single-molecule fluorescence resonance energy transfer, and single-molecule fluorescence resonance energy transfer offer promising avenues to resolve these questions and reveal how specialized ribosomes contribute to adaptive gene expression.

## Teaser

Ribosomal heterogeneity in *Saccharomyces cerevisiae*, driven mostly by protein paralogs and regulatory rancRNAs, plays a critical role in stress adaptation by modulating ribosome composition and translation efficiency.

## Introduction—specialized ribosomes

The biogenesis of a ribosome is a highly precise process during which ribosomal RNAs (rRNAs) and ribosomal proteins (RPs) are rigorously assembled. In yeast, the ribosomal 40S subunit contains 33 RPs and 18S rRNA, while the large ribosomal subunit 60S is composed of 46 RPs and three distinct rRNAs, namely 5S, 5.8S, and 25S (Rabl et al. 2011, Klinge et al. 2011). Responsible for translating proteins, the ribosomes are extremely abundant in all cells. It is estimated that rapidly growing *Saccharomyces cerevisiae* produce and export up to 4,000 ribosomes per minute per cell to the cytoplasm, amounting to ∼2–4 × 10^5 (Warner 1999, Shore and Albert 2022).

In the years after the discovery of ribosomes in the 1950s, small differences in their sizes and shapes observed under an electron microscope led scientists to theorize about the heterogeneity of ribosomes. In 1958, Francis Crick proposed a model called the “one gene - one ribosome - one protein hypothesis,” implying that a distinct type of ribosome synthesizes each protein. This theory was quickly abandoned, and since then, ribosomes have been considered undifferentiated and uniform macromolecular machines whose sole function was to bind mRNA and translate proteins (Brenner et al. 1961, Gros et al. 1961, Palade 1975, Genuth and Barna 2018, Emmott et al. 2019). The first hints that ribosomes may not be a homogenous group of macromolecules came from reports describing differential expression of particular ribosomal components in various organisms. Currently, due to the constant influx of new data, the ribosome is no longer viewed as a passive, uniform machinery but instead as a dynamic macromolecular complex that fulfills precise and specialized roles in the cell (Table 1).

**Table 1.** Sources and functional consequences of ribosome heterogeneity in yeast.

Source of ribosome heterogeneity	Description	Examples in *S. cerevisiae*	Functional consequences	Key references
**RP paralogs**	Presence of duplicated paralogous RPGs encoding identical or slightly different proteins (1–5 amino acids difference).	59 RPs encoded by two paralogous genes; e.g. RPL7A/RPL7B, RPS9A/RPS9B.	Functional specialization, different phenotypes on deletion, impact on drug resistance and cell fitness.	Parenteau et al. (2015), Ghulam et al. (2020), Pietras et al. (2024)
**Introns and UTRs**	Introns enriched in RPGs^a^ regulate splicing and expression; UTR sequences affect transcript variants.	RPS22B (5′ UTR intron regulates expression under osmotic stress); RPS9 paralogs show differential splicing.	Cell-to-cell expression heterogeneity, differential stress responses, fine-tuned RP expression.	Parenteau et al. (2011), Roy et al. (2020), Lukačišin and Bollenbach (2019), Petibon et al. (2016)
**RP stoichiometry and composition**	Variation in abundance and incorporation of RP paralogs into ribosomes depending on growth or stress conditions.	Changes in paralog ratios (e.g. RPL22/eL22, RPS28/eS28 under salt and drug stress); dynamic remodeling during heat shock or salinity stress.	Modulates ribosome function and specificity, influences translation under environmental stresses.	Slavov et al. (2015), Ghulam et al. (2020), Sun et al. (2021), Pietras et al. (2024)
**Post-translational modifications of RPs**	Phosphorylation, methylation, ubiquitination, hydroxylation, acetylation, SUMOylation of RPs.	RPS23 hydroxylation modulates translational accuracy; RPS6 phosphorylation regulated by TORC1; RPL28 ubiquitination varies with cell cycle.	Affects translation fidelity, ribosome stability, localization, and synthesis rates.	Lee et al. (2002), Spence et al. (2000), Al-Hadid et al. (2016), Yerlikaya et al. (2016)
**Ribosome-associated factors**	Proteins binding to ribosomes influencing translation selectivity and efficiency.	Asc1 (RACK1 homolog) modulates translation of stress-related mRNAs; Scp160 scaffolds mRNA localization; RAC and Ssb chaperones assist co-translational folding.	Selective translation, stress response modulation, protein folding; changes in ribosome profiles under stress.	Pfund et al. (1998), Baum et al. (2004), Sezen et al. (2009), Koplin et al. (2010), Thompson et al. (2016)
**rRNA nucleotide polymorphism**	Multiple rDNA repeats with polymorphisms (SNPs, indels) found in 26S, 18S, 5.8S, and 5S rRNAs; polymorphisms clustered mainly in expansion domains.	150–200 tandem repeats on chromosome XII; 227 polymorphic sites found across strains; partial SNPs present.	Potential influence on ribosome function via sequence variation, but no stress-induced changes reported in yeast yet.	James et al. (2009), Kwan et al. (2013)
**Post-transcriptional rRNA modifications**	12 classes of modifications at 112 positions, mainly pseudouridylation and 2′-O-methylation; modification patterns stable under stress.	Absence of 2′-O-methyl group at A100 in 18S rRNA in actively translating ribosomes; no epitranscriptomic changes upon oxidative or thermal stress.	Modifications affect rRNA structure, ligand binding, translational fidelity; some ribosomes lack 2′-O-methylation at A100 in 18S rRNA.	Baudin-Baillieu and Namy (2021), Begik et al. (2021)
**Ribosome-associated noncoding RNAs (rancRNAs)**	Small ncRNAs (18–35 nt) derived from rRNA, tRNA, snoRNA, and mRNA associate transiently with ribosomes; binding is often stress-dependent.	Examples include: 23-nt fragment from 25S rRNA; tRNA-derived fragments (tDRs) like from tRNA-His-GTG; snoRNA-derived fragments (sdRNAs) such as sdR67, sdR83, sdR128; 18-mer ncRNA from TRM10 mRNA regulating translation under salt stress.	rancRNAs modulate translation by inhibiting protein synthesis, regulating ribosome function under stress (e.g. salt stress, starvation); may form specialized ribosome pools.	Zywicki et al. (2012), Pircher et al. (2014), Bąkowska-Żywicka et al. (2016), Mleczko et al. (2019)

a RPGs, RP genes.

## Ribosomal proteins

In the *S. cerevisiae* genome, 59 RPs are transcribed from duplicated paralogous genes. Indeed, in this species, 118 of the 137 RPGs are duplicated (Wapinski et al. 2010, Woolford and Baserga 2013, Parenteau et al. 2015). The occurrence of paralogous RPGs in yeast was initially thought to result from genome doubling that happened early in evolution and became fixed later. However, Marcet-Houben and Gabaldón (2015) suggested that the first step must have been a cross between two closely related species, and the genome doubling itself was due to the need to restore fertility. The functional paralogs (ohnologs) are thought to have evolved in their function through mutation or differences in expression patterns following a hybridization event (Parenteau et al. 2011, Parenteau et al. 2015).

Of the 79 yeast RPs, 59 are encoded by two paralogous genes (Simoff et al. 2009). Twenty-one duplicated RPGs in yeast encode identical proteins, while 38 pairs of RPs differ from each other by typically 1–5 amino acids, which suggests the possibility of performing various functions by these paralogs. These assumptions are further supported by the results of studies in which the deletion of yeast ohnologs resulted in the generation of distinct phenotypes, suggesting that the RPs may have evolved specialized functions (Komili et al. 2007, Parenteau et al. 2011).

### The role of introns and UTRs

In the *S. cerevisiae* genome, fewer than 5% of genes (280 in total) contain introns. RPGs are particularly intron-rich, with 94 RPGs harboring introns, corresponding to 80% of the 118 duplicated RPGs but only 32% of the unique RPGs (Parenteau et al. 2019). Wide-scale intron-deletion studies revealed that half of the yeast introns accumulate and/or are under-spliced in the stationary phase of growth, impacting the regulation of RPGs and cellular response to stress and starvation (Parenteau et al. 2015).

Parenteau et al. (2011) analyzed the influence of the introns present within RP mRNAs on the production and function of yeast ribosomes and demonstrated that intron deletion in RPGs generally does not cause growth defects in rich media but significantly affects expression levels of key ribosome biogenesis factors. For example, deletion of the intron in *RPL2A* reduced cell fitness drastically, and intron deletion in duplicated genes often affected only one paralog, implying functional specialization of RP paralogs in fitness and drug resistance. Initial functional analysis of intron-dependent regulation of RPGs included 8 different carbon sources, 16 drugs, and 3 different temperatures. It showed that out of all tested variables, only five drugs related to protein synthesis induced growth defects in mutant strains carrying intron deletions in RPGs. Additionally, the authors showed that 17% of all intron deletions decreased cell fitness, and 25% increased cell fitness in rich media, compared to the wild-type strain. Moreover, 36% of the large subunit genes affecting fitness are implicated in bridging the two subunits; e.g. deletion of the intron from the *RPL2* gene reduced fitness to 14% compared to wild-type yeast. Intron deletion from RPG encoding *RPS29* reduced fitness to 16% of the wild-type level. Notably, the vast majority of intron deletion mutations caused effects only on one copy of the duplicated RPGs. In the case of the *RPL23A*/*RPL23B* ohnolog pair, the deletion of the intron in *RPL23A* only reduced cell fitness to 20% of wild-type, while no observable effect was visible for *RPL23B*. This observation clearly suggests that at least some functional RP paralogs play exclusive roles in drug resistance and cell fitness (Parenteau et al. 2011).

A clear example of intron-mediated regulation is the *RPS22B* gene, which contains a 5′ UTR intron with regulatory elements controlling its own splicing via feedback mechanisms (Hooks et al. 2016, Roy et al. 2020). Under osmotic stress (LiCl, NaCl, and KCl), retention of this intron generates cell-to-cell heterogeneity in RPS22B expression, directly influencing fitness: cells with low RPS22B levels survive prolonged starvation better, while those with higher expression perform better after short-term starvation (Lukačišin and Bollenbach 2019). This highlights how intron retention can serve as a cis-regulatory mechanism that fine-tunes RPG expression in response to environmental conditions.

Other examples include the RPS9 paralogs, where RPS9B exhibits preferential expression over RPS9A as a result of asymmetric paralog-specific splicing exerted by their respective intronic sequences and the 3′ untranslated region (Petibon et al. 2016). Under standard growth conditions, the expression ratio between RPS9A and RPS9B is maintained. Under stress, intron deletion disrupts normal regulation, leading to constitutive upregulation of RPS9A (Parenteau et al. 2011) and masking stress responses (Petibon et al. 2016). Such examples emphasize the critical role of introns in modulating paralog-specific expression and cellular adaptation.

In contrast, the expression of nonintron-encoding RPGs (niRPGs) depends on ohnolog-specific regulatory sequences present in promoters, UTRs, and transcription-termination sequences (Parenteau et al. 2015). One such example is the ohnolog pair *RPL8A*/*RPL8B*, where *RPL8A* has two clearly distinguishable 3′ends: T1 generated by canonical polyadenylation-dependent transcription termination, and T2 generated through cleavage by Rnt1p Rnase III, while *RPL8B* has only one 3′end. Deletion of the Rntp1 cleavage site resulted in the generation of only one long *RPL8B* transcript and 6 *RPL8A* transcripts differing in 5′ ends. It has been shown that weak transcription-termination sites mediate the heterogeneity of RPL8A transcripts.

Additionally, functional redundancy evaluation of niRPGs on cell growth and fitness showed that the deletion of many niRPG ohnologs impairs growth and stress resistance. Analyses of the influence of 14 drugs on the expression of niRPG ohnologs revealed that the majority of tested conditions affected cell growth in an ohnolog-specific manner. What is more, the authors showed that RPL8A acts as a dedicated hygromycin B stress response gene (Parenteau et al. 2015).

### RP stoichiometry and composition

Ribosome heterogeneity can arise from alterations in the relative abundance of RPs, leading to differences in RP stoichiometry and resulting in subsets of ribosomes with reduced incorporation of specific RPs. An observation that the RPs whose levels differ the most among the different growth conditions (glucose or ethanol as carbon source) are located on the surface of the yeast ribosomes, as exemplified by RPL17A and RPL17B ohnologs, has been made by Slavov et al. (2015). The study demonstrated that RP stoichiometry is influenced by two key factors: the number of ribosomes per mRNA, and the carbon source in the growth medium. This correlation pattern was consistently observed under both ethanol- and glucose-based carbon sources.

In *S. cerevisiae* Ghulam et al. (2020) observed that the ratio of 16 ohnolog pairs was altered in at least one stress condition tested (NaCl or hygromycin), influencing cell fitness. Analysis of protein incorporation into ribosomes revealed that the ratio between major and minor paralog was decreased for RPL22, RPL8, RPL9, RPS28 for hygromycin, and RPL14, RPL4 for salt stress. At the same time, RPL21 and RPL7 were modified in both stress conditions. Moreover, two paralog pairs were presented with inverted ratios: RPL34 in response to hygromycin and NaCl, and RPS1 in response to NaCl. Additional analyses revealed a preferential selection of ribosomes carrying major paralog of RPL14, RPL26, RPS9 pairs, and minor paralog in the case of RPL22, RPL7, RPL8, RPL9, and RPS28 for protein biosynthesis under at least one stress condition tested. These shifts correlate with adaptive responses, indicating functional consequences of paralog variation. These observations were further substantiated by Parenteau and coworkers, who described the modification of ohnolog pairs’ ratios in a drug-dependent manner (Parenteau et al. 2015).

In a recent study, our group analyzed the RP heterogeneity of ribosomes isolated from yeast subjected to 10 environmental stresses using liquid chromatography/high-resolution mass spectrometry (Pietras et al. 2024). Statistically significant differences in the abundance of 14 RPSs and 8 RPLs localized mostly at the outer core of ribosomes (similarly to Slavov et al. 2015) were detected, with the most prominent changes taking place during high salinity (NaCl) stress, where the levels of three of 15 ohnologs were increased (RPL1, RPL6, and RPL42). The amounts of the remaining 12 ohnologs were decreased, with the most significant change detected for RPS30. Surprisingly, increased amounts of nine RPs (1.17–1.46 fold change when compared to the control conditions) were detected under several stress conditions. Moreover, under stress conditions, the paralog ratios of four RPs (RPL16, RPS1, RPS7, and RPS9) changed significantly and were present within yeast ribosomes mostly in the form of paralog B. A 33.46% increase in the ratio of paralog A to paralog B during heat shock was detected for small ribosomal subunit RPS7. Paralog A/B ratios also changed for RPL14, RPL16, RPL22A, RPS1A, RPS9A, and RPS29A under different stress conditions (Pietras et al. 2024). These significant changes in paralog ratios observed during multiple stresses in *S. cerevisiae* emphasize that ribosomes dynamically adjust paralog composition in response to environmental cues. The dynamic nature of ribosomes in *S. cerevisiae* was further supported by cryo-EM data showing selective depletion of proteins such as RPS1 and RPL16 during shifts in growth conditions, suggesting ribosome remodeling rather than *de novo* assembly of specialized ribosomes (Sun et al. 2021).

Regarding paralog expression dosage, Palumbo et al. (2017) demonstrated that in *S. cerevisiae*, RPL7A is expressed more highly than RPL7B (independent of genomic context), and that total RPL7 protein levels rather than isoform identity determine phenotypic outcomes such as stress sensitivity to tunicamycin, ASH1 mRNA localization, and Ty1 retrotransposon mobility. The role of introns in RPG expression is often linked to broader cellular processes, but mechanistic understanding remains limited and requires further study. The genes of two differentially expressed in wild-type yeast RPs RPL7A and RPL7B contain paralogous C/D box snoRNA genes, snR39 or snR59 (encoded in the second intron) that function as guide RNAs for 29-O-methylation of residue A807 in the large subunit rRNA (Komili et al. 2007, Piekna-Przybylska et al. 2007, Palumbo et al. 2017). Shamsuzzaman et al. (2023) reported that disruption of ribosome biogenesis or translation leads to distinct and specific effects on RPG expression during nucleolar and translation stresses in *S. cerevisiae*. Repression of RPL4 synthesis initially led to a decrease in mRNA levels of other RPs during the first 2 hours, followed by a subsequent upregulation. In contrast, an initial induction of RPs’ transcription was followed by their repression, leading to the conclusion that RPGs were induced during nucleolar stress and repressed during translation stress. Moreover, the authors showed that the relative expression of RPGs of paralogue RP pairs changed more than 2.5-fold for RPS8, RPS28, RPL6, RPL18, RPL22, RPL30, and RPL33. The remaining RP paralogues exhibited only modest changes in expression under the analyzed stress conditions. Such significant shifts in expression of several paralog pairs under ribosome biogenesis or translation stress highlight complex regulation during stress responses. In another study (Ferretti et al. 2017), it has been shown that RPS26 is necessary for efficient translation of mRNAs containing adenosine at the position −4 of the Kozak sequence. Conversely, ribosomes lacking Rps26 (ΔRps26) preferentially translate mRNAs with a guanosine at this position. Pathway enrichment analysis of transcripts associated with ΔRps26 ribosomes revealed a significant clustering within the Hog1 and Rim101 signaling pathways, which are central to the cellular response to osmotic (high salt) and alkaline pH stress, respectively. Importantly, exposure to high salt or pH conditions induces the formation of ribosomes lacking Rps26 (ΔRps26), thereby enabling the selective translation of mRNAs containing noncanonical nucleotides at the − 4 position of the Kozak sequence (Ferretti et al. 2017).

In summary, selective examples where paralog expression modulates ribosome composition and cell fitness better illustrate the functional importance of RP paralogs than broad lists of expression changes. The dynamic and adaptive nature of ribosome composition is increasingly recognized as a critical layer of translational regulation.

### RP chemical modifications

It has been postulated for some time that another layer of ribosomal heterogeneity can be introduced via post-translational modifications (PTMs) to RPs, since these proteins are subjected to many modifications such as phosphorylation, ubiquitination, methylation, acetylation, hydroxylation, or SUMOylation (Lee et al. 2002, Simsek and Barna 2017). Although multiple examples of differential PTMs of RPs have been identified, their functional significance remains largely unclear; however, they are hypothesized to influence the stability, subcellular localization, or interaction networks of the modified proteins. Small subunit RP RPS23p, located in close proximity to the ribosomal decoding center, undergoes hydroxylation on Pro-64 residue as a result of hypoxic stress, catalyzed by a hydroxylase belonging to the Fe(II) and 2-oxoglutarate dependent oxygenases family. The authors demonstrated that hydroxylation of RPS23p modulates translational accuracy in a manner dependent on the specific stop codon context, either enhancing or reducing fidelity.

Nine RPs have been identified as methylated in *S. cerevisiae*: four from the small ribosomal subunit (RPS2, RPS3, RPS25AB, and RPS27AB) and five from the large (RPL1AB, RPL3, RPL12AB, RPL23AB, and RPL42AB) (Chern et al. 2002, Lee et al. 2002, Porras-Yakushi et al. 2005, Lipson et al. 2010, Webb et al. 2010 a, 2010b, Young et al. 2012), with seven modified residues exposed to the cytoplasm, two at the subunit interface, and three embedded within the rRNA core (Clarke 2013). It has been shown that methylation of RPL3 at His243, which takes place on ribosome-bound, rather than free RPL3, plays an important role in translation elongation (Al-Hadid et al. 2016). This protein, located at the peptidyl-transferase center, plays a central role in coordinating the decoding, peptidyl transfer, and translocation steps during translation elongation. Cells expressing the RPL3–H243A mutant exhibited impaired translation elongation, leading to reduced translational fidelity (Al-Hadid et al. 2016).

Budding yeast RPS6 is encoded by two independent open reading frames, RPS6A and RPS6B, which arose from genome duplication. Two phosphorylation sites (Ser232 and Ser233) exist at the C-terminal region of RPS6A and RPS6B. RPS6 was one of the first RPs found to undergo phosphorylation (Kabat 1970, Gressner and Wool 1974). RPS6 phosphorylation is differentially regulated downstream of TORC1 via Ypk3 and TORC2 via Ypk1 and Ypk2. TORC1 additionally appears to regulate RPS6 dephosphorylation via Glc7/Shp1. Genetic and pharmacological studies have provided mechanistic insights into the regulation of TORC1 signaling in response to nutrients. In yeast *S. cerevisiae*, nutrients rapidly induce RPS6 phosphorylation in a TORC1-dependent manner. Moreover, Yeast RPS6 is phosphorylated after transfer of a stationary culture to fresh nutrient medium, as well as at an early stage of germination, and as in other eukaryotes, the protein is dephosphorylated during heat shock (Szyszka and Gasior 1984, Jakubowicz 1985, Meyuhas 2008, Gonzalez et al. 2015, Yerlikaya et al. 2016).

Another RP modification with a clear link to modulating protein synthesis is ubiquitination. In yeast, the level of RPL28 (localized in the peptidyl transferase center of the ribosome) ubiquitination fluctuates throughout the cell cycle, exhibiting low abundance during the G0 and G1 phases and increasing during the S phase. Ribosomes with polyubiquitinated RPL28 carry out protein synthesis at a higher rate *in vitro* compared with ribosomes with monoubiquitinated RPL28 (Spence et al. 2000). Moreover, it has been shown that in *S. cerevisiae*, exposure to H₂O₂ induces strong and specific K63-linked polyubiquitination of numerous proteins, including many RPs. This mechanism helps stabilize monosomes and polysomes and supports the translational response to oxidative stress (Silva et al. 2015).

### Ribosome-associated factors

Ribosome-associated factors play a central role in generating functional ribosomal heterogeneity in *S. cerevisiae*, influencing not only translational efficiency but also selectivity toward specific mRNA subsets. These factors interact dynamically with translating ribosomes and contribute to distinct ribosomal populations with specialized translational functions. One of the most studied ribosome-associated proteins in yeast is Asc1 (the homolog of mammalian RACK1), a WD40-repeat protein located on the head of the 40S subunit near the mRNA exit tunnel. Asc1 influences translation of specific mRNAs involved in stress response and cell signaling, and its absence alters the translational profile without affecting global protein synthesis (Sezen et al. 2009, Thompson et al. 2016). In addition, Asc1 is necessary for efficient translation of mRNAs with short ORFs in *S. cerevisiae* (Thompson et al. 2016). Similarly, the Scp160 RNA-binding protein, which interacts with polysomes via multiple RNA-binding heterogeneous nuclear ribonucleoprotein K-homology domains, is proposed to act as a scaffold coordinating mRNA localization and translation. It is located in close spatial association with translation elongation factor 1A and the Asc1p protein on the ribosome. Its association with ribosomes is thought to support selective translation during polarized growth (Baum et al. 2004).

Chaperone systems such as the ribosome-associated complex (RAC), composed of Zuo1 and Ssz1, and the Ssb1/2 Hsp70 chaperones, also contribute to ribosomal specialization. These factors bind nascent polypeptides emerging from the ribosome and are involved in co-translational protein folding. Importantly, RAC–Ssb association with the ribosome is not uniform but varies with growth conditions and translational demands, indicating potential specialization of ribosome–nascent chain complexes (Pfund et al. 1998, Koplin et al. 2010). Deletion of RAC components leads to altered ribosome profiles, translational defects, and increased protein aggregation.

Recent proteomic and ribosome profiling approaches have revealed dozens of noncanonical ribosome-associated proteins, including metabolic enzymes and RNA helicases, that associate with specific ribosome subpopulations (Fleischer et al. 2006, Shiber et al. 2018). These interactions further expand the potential for ribosomal functional specialization by linking translation to metabolic state, stress response, or RNA processing. Together, these findings demonstrate that ribosome-associated factors in yeast contribute to a diverse and dynamic landscape of ribosomal complexes, allowing for the selective translation of specific mRNAs in response to physiological and environmental conditions.

## Ribosomal RNAs

Recently, rRNA nucleotide polymorphism has been shown to be the source of ribosomal heterogeneity in both prokaryotic organisms (Song et al. 2019) and eukaryotic cells (Parks et al. 2018). In the marine pathogenic bacterium *Vibrio vulnificus*, it was observed that incorporation of the most variable rRNA, encoded by the *rrnI* operon, into ribosomes led to rapid adaptation to temperature and nutrient stresses due to the preferential translation of a specific subset of mRNAs (Song et al. 2019). What is more, rRNA ribosomal heterogeneity has been observed in polysomes isolated from mouse mammary epithelial cells, confirming that genomically encoded rRNA variants are present in actively translating ribosomes (Song et al. 2019). Such observations support the hypothesis that different rRNA variants incorporated into ribosomes may influence ribosome function in ways that affect gene expression and cell physiology.

### rRNA-encoding genes

To date, there are no literature reports on ribosome heterogeneity regarding rRNA molecules in the yeast *S. cerevisiae* occurring under the influence of stress conditions. Nevertheless, some changes in the rDNA sequences have already been described. The high demands of cells for continuous protein biosynthesis are reflected in the numerous copies of rRNA-encoding genes existing in many repeats in the genomes of all organisms. In *S. cerevisiae*, rDNA arrays of over 1 MB in size typically contain 150–200 tandem repeats and occupy 60% of chromosome XII. Each repeat (9.1 kb sequence) encodes four rRNA genes: 26S, 18S, 5.8S, and 5S, as well as two internal transcribed spacers: ITS1 and ITS2, and a large intergenic spacer (IGS) (James et al. 2009, Kwan et al. 2013). Large-scale analysis of over 35 Mbp of rDNA sequencing data of the *Saccharomyces* Genome Resequencing Project (www.sanger.ac.uk/Teams/Team118/sgrp, 34 strains) has led to the alignment of rDNA-specific shotgun reads from a wide variety of sources, including baking, brewing, laboratory, pathogenic, pro-biotic, and environmental, and from numerous locations around the world to rDNA consensus sequence of the *S. cerevisiae* reference strain (S288c). The level of variation within individual rDNA arrays was found to be surprisingly high, ranging from 10 to 76 polymorphisms between strains. In total, James and coworkers (James et al. 2009) identified 227 polymorphic sites within rDNA arrays: 44 in the rRNA-encoding genes, 27 in the ETS region, 11 in the ITS region, and 146 in the nontranscribed IGS region. Of all base substitution types, transitions were the most abundant form of mutation detected in rDNA arrays, representing 71% (162 of 227 sites). Additionally, as many as 29 out of 40 polymorphisms identified in 26S and 18S rRNA-encoding genes were found in expansion regions, which evolve more quickly than core regions because they are under much smaller functional constraints, or in single-stranded and loop regions. The most affected region of 26S rRNA was the D7 domain, containing 11 polymorphisms. Additionally, the authors detected differences in the length of analyzed rDNA arrays ranging from 9083 to 9147 bp in various strains, which is suggestive of indel occurrences (James et al. 2009). Importantly, many of the rDNA array polymorphisms identified for each strain did not occur on every sequence read from that strain, which led the authors to name the changes as partial SNPs.

### Post-transcriptional rRNA modifications

Apart from differences in rRNA sequences, post-transcriptional rRNA modifications may also be the source of ribosome heterogeneity. In *S. cerevisiae* rRNAs, 12 different classes of modifications in as many as 112 positions have been reported, with uridine-to-pseudouridine isomerization and methylation at the ribose 2′OH being the most common. Significantly, these modifications influence secondary and tertiary structures of rRNAs and interactions with tRNA, mRNA, and proteins (Jack et al. 2011, Abou Assi et al. 2020, Baudin-Baillieu and Namy 2021, Lin et al. 2021). Recently, it has been shown that a substantial amount of translating ribosomes in actively growing *S. cerevisiae* lack a 2′-O-ribose methyl group at nucleotide A100 in the 18S rRNA; however, the role of these ribosomes is still not known (Buchhaupt et al. 2014). Several other studies indicated that differential pseudouridilation of rRNAs leads to defects in ribosomal ligand binding and impaired translational fidelity (Bellodi et al. 2010, Jack et al. 2011). However, the expectation that cells might be able to generate compositionally distinct ribosomes in response to environmental changes still needs to be confirmed, as in a recent study, neither oxidative nor thermal stresses caused changes in the ribosomal epitranscriptome in *S. cerevisiae* (Begik et al. 2021).

### Ribosome-associated noncoding RNAs

Noncoding RNAs (ncRNAs) are RNA molecules that do not undergo translation into proteins. Novel classes of small ncRNAs (18–35 nucleotides in length) continue to be identified, with well-established roles in development, stress responses, and disease processes. Increasingly, consistent evidence also highlights their fundamental regulatory functions in gene expression across multiple levels. Although ncRNAs are not structurally integral components of the ribosome, transient associations between ncRNAs and ribosomes—reported by several research groups—suggest that such interactions may contribute to ribosomal heterogeneity and influence translational dynamics.

Although it has long been known that signal recognition particle RNAs and transfer-messenger RNAs associate with the ribosome, it has only recently been shown that such an interaction is also possible for shorter noncoding RNAs (Fu et al. 2011, Janssen and Hayes 2012, Jomaa et al. 2017, Becker et al. 2017). It was not until 2012 that the first report on the interaction of short ncRNAs with ribosomes was published (Zywicki et al. 2012). The authors analyzed small ribosome-associated RNA libraries (size range ∼15–500 nucleotides) derived from *S. cerevisiae* grown in 12 different conditions. They identified multiple novel stable RNA molecules differentially processed from well-known ncRNAs, like rRNAs, tRNAs, or snoRNAs. The most abundant classes of RNA identified in the library were small RNAs derived from rRNAs. The presence of a 23-nt fragment from the 5′-part of 25S rRNA has been verified experimentally with the means of quantitative real-time PCR, and established to be present in 12 yeast growth conditions comprising heat, cold, high salinity, high or low pH, UV exposure, hyper- or hypoosmotic conditions, amino acid or sugar starvation, and anaerobic and normal growth. Apart from rRNAs, the most abundant classes of small noncoding RNAs identified in their library originated from tRNAs (41 tRNA-derived fragments, tDRs) and snoRNAs (snoRNA-derived fragments, sdRNAs). tRNA cleavage occurred in the anticodon loop, as well as other breakage points, like in the D- and T-loop regions, and the resulting fragments were characterized by different stability, depending on the conditions, as reported for two stable processing products derived from tRNA-His during amino acid and sugar starvation. Only one tDR derived from the 3′-end of tRNA-Ser was stable in the tested conditions, while those originating from the 5′-end were most likely degradation products. Stable snoRNA fragments were also detected among molecules directly interacting with ribosomes under most growth conditions. snoRNA 128 signals on northern blots were detected in the polysomal fractions, suggesting that snoRNAs are associated with translating ribosomes in yeast (Zywicki et al. 2012).

In addition, it has been shown that mRNA-derived 18-nucleotide-long ncRNA identified in the same screen (Zywicki et al. 2012) can downregulate translation in *S. cerevisiae* by targeting the ribosome (Pircher et al. 2014). This ncRNA originates from tRNA methyltransferase TRM10 mRNA, and constitutes a fragment located 28 nucleotides downstream of the translation start site. Under nonstressful conditions, >80% of this 18-mer binds to the large subunit of 80S ribosomes, whereas in yeast cells subjected to high salinity stress, it binds to polysomes. Interestingly, no growth defects were observed in cells expressing the nontranslatable version of TRM10 mRNA. Still, cells failed to resume growth when mutations were introduced in two or three synonymous codons into the *TRM10* gene, despite active translation of this tRNA methyltransferase. This observation led the authors to conclude that binding the 18-mer RNA to polysomes is necessary for yeast cells to grow under salt stress conditions. Importantly, this interaction is specific for high salt stress only, as it did not change yeast growth when subjected to temperature stresses. What is more, almost complete inhibition of translation was observed upon the introduction of 18-mer to yeast spheroplasts and in *in vitro* translation experiments (Pircher et al. 2014).

To further characterize tRNA-derived small RNAs identified in rancRNA library (Zywicki et al. 2012), Bąkowska-Żywicka et al. chose six molecules with robust read coverage (Bąkowska-Żywicka et al. 2016). Based on tDR/ribosome saturation assays, the authors determined that the ribosome began to saturate with tDRs when the ribosome:tDR ratio reached 1:1 and achieved full saturation at a ratio of 1:2. Additionally, using *in vitro* competition binding assays they were able to determine that all tested yeast 3′-tDRs occupy the same ribosomal binding site, which is different than the canonical ribosomal tRNA binding sites (A and P sites), as well as 5′-tDR–His–GTG binding site. The authors observed that tDR/ribosome interaction was stress-dependent, with binding efficiency in the range between 8% and 45% for most tested tRNA fragments. The highest binding (62%–100%) was noted for 5′- and 3′-parts of tRNA–His–GTG in yeast subjected to low pH conditions and amino acid or sugar starvation. It has also been determined that these tDRs decrease protein biosynthesis to 24%–35% in most tested conditions, with 95% and a total inhibition recorded for tRNA–His–GTG tDRs under low pH, and amino acid or sugar starvation, respectively (Bąkowska-Żywicka et al. 2016).

Based on the highest read coverage observed in ribosome-associated small RNA sequencing data in *S. cerevisiae* (Zywicki et al. 2012), Mleczko et al. (2019) selected three snoRNAs and their corresponding sdRNAs for precise quantitative analysis. First, they determined that both full-length snoRNAs and sdRNAs were present in ribosome-containing fractions, and this association was strongly stress-dependent. The authors were also able to quantify the levels of particular molecules precisely. The highest concentrations of snR67 and snR83 were recorded in yeast subjected to high pH, with nearly 300,000 and 160,000 copies/μl, respectively, and of snR128 during heat shock (∼280,000 copies/μl). Moreover, the authors determined that the stress-dependent association of full-length snoRNAs and small sdRNAs with yeast ribosomes is independent. To analyse the possible functions of the ribosome-associated sdRNAs during protein biosynthesis, *in vitro* translation experiments in yeast grown in optimal growth conditions were performed, using the total endogenous mRNA pool as template and ^35^S-methionine incorporation into proteins to measure the translation efficiency. As reported, the introduction of 500 pmoles of sdR67 or sdR83 lowered the translation to 40% and 75%, respectively, while as little as 10 pmoles of sdR128 was enough to reduce protein biosynthesis observably. These observations were further confirmed in in vivo *S. cerevisiae* translation experiments (Mleczko et al. 2019).

Although ncRNAs are not permanent structural components of the ribosome, studies presented above demonstrated their presence in ribosomal fractions, including polysomes, under diverse growth and stress conditions (Zywicki et al. 2012, Bąkowska-Żywicka et al. 2016, Mleczko et al. 2019). Collectively, these findings support a model in which ribosome-associated ncRNAs contribute dynamically to the formation of specialized ribosomal pools with distinct translational capacities. However, the precise mechanisms by which these ncRNAs influence ribosome composition and function remain to be fully elucidated, representing an important avenue for future research.

## Future perspectives

**Figure 1.**
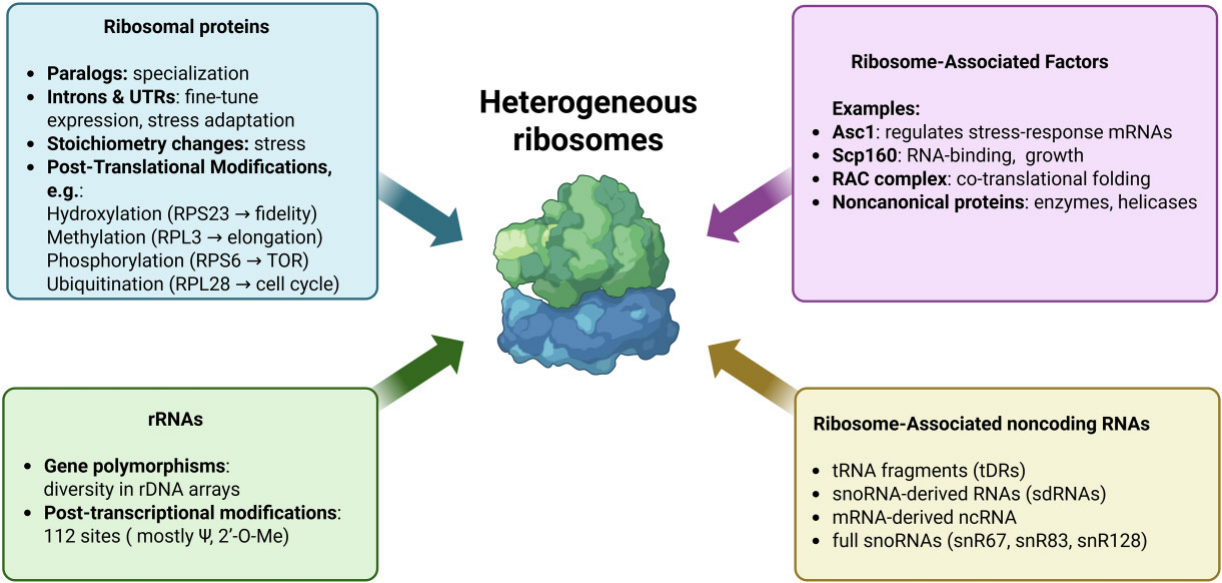
The general overview of possible sources of ribosomal heterogeneity.

The study of specialized ribosomes and their heterogeneity has rapidly evolved from the “one gene - one ribosome - one protein” concept into a pivotal area of research. In the past years, we have witnessed increasing amounts of experimental data on distinct, in terms of composition, translationally active ribosomes in various organisms, from *Escherichia coli* to humans. There are many potential sources of this heterogeneity: RPs, their paralogs, rRNAs, RP, and rRNA modifications, as well as noncoding RNAs and ribosome-associated factors (Fig. 1). The scientific community agrees that considerable ribosome heterogeneity exists across most, if not all, cell types. A subset of this variability will likely result in functional differences defining so-called “specialized ribosomes.”

However, despite substantial evidence supporting the existence of ribosome diversity, the limited availability of functional data on the specific roles of distinct ribosomal subpopulations remains a significant factor contributing to the continued controversy in the field. Competing views on specialized ribosomes center around whether ribosomes are uniform machines or can vary in composition to regulate translation of specific mRNAs. One view argues for functional specialization, which can influence selective mRNA translation. The opposing view suggests observed differences may reflect dynamic cellular states or experimental artifacts, not true specialization. The science review by Mills and Green (2017) highlights evidence supporting specialization, including how mutations in specific RPs (e.g. RPS19 in Diamond–Blackfan anemia) lead to tissue-specific diseases. These mutations may impair translation of particular transcripts essential for certain cell types, suggesting that ribosomes with distinct compositions can have specialized roles in gene expression. A prominent viewpoint in this review is that many tissue-specific phenotypes result from the variable expression or incorporation of RP A/B isoforms. These isoforms can subtly alter ribosome composition, potentially modulating its functional properties and enabling selective translation of specific mRNAs critical for particular cell types. This mechanism provides a plausible explanation for how mutations in RPs lead to distinct tissue-specific diseases by affecting the translation of key transcripts in those tissues.

Importantly, specialized ribosomes can preferentially translate specific subsets of mRNAs, contributing to gene expression regulation at the translational level. This selectivity often arises through differential affinity or interactions with mRNA features such as specific 5′ UTRs, IRES, uORFs, or RNA-binding proteins. For example, ribosomes lacking certain RPs (e.g. RPL38) have been shown to selectively translate Hox mRNAs during development, influencing tissue patterning (Kondrashov et al. 2011). Similarly, heterogeneous ribosomes with distinct RP compositions or rRNA modifications can recognize specific RNA motifs or structures, enabling preferential translation of mRNAs involved in stress responses or cell differentiation (Slavov et al. 2015, Shi et al. 2017). Additionally, post-transcriptional modifications and ribosome-associated factors can modulate mRNA selection, further fine-tuning translational specificity (Xue and Barna 2012).

At the same time, as our understanding of ribosome complexity deepens, exciting future perspectives arise. Some of the questions that need to be answered are: (i) what is the extent of ribosomal heterogeneity in various cells and organisms, and how it influences the activity and specificity of the ribosomes; (ii) what is the biological significance of ribosome heterogeneity, i.e. the functional consequences of ribosomal specialization and how they translate to particular phenotypes. Notably, current research suggests that such specialization may be context-dependent, manifesting under specific conditions such as cell-type-specific translational programs, developmental stages, or environmental stimuli, wherein distinct ribosomal subpopulations may be assembled and targeted to particular cellular compartments.

Ribosome heterogeneity presents a plethora of fascinating scientific questions that warrant investigation. In our view, beyond the essential analysis of rRNA and RP post-transcriptional modifications, as well as the compositional diversity of rRNA and proteins across different organisms and physiological conditions, several additional research pathways worth considering are: (i) analysis of ribosomal specialization in organisms growing in extreme conditions, as well as during disease; (ii) given that some RPs, including paralogs, function as moonlighting proteins, it would be interesting to investigate how they contribute to specific cellular processes, including stress responses; (iii) investigation of mechanisms through which rancRNAs interact with ribosomes, especially under various stress conditions; and (iv) studying of how ribosomes selectively translate subsets of mRNAs that are critical for cell survival under adverse conditions via stress-dependent ncRNA/ribosome interactions. These research directions may be greatly facilitated by applying ribosome profiling, ribosome fractionation, single-cell high-throughput techniques, and, ultimately, high-throughput single-ribosome methods. Changes in initiation or elongation rates may be studied by applying selective translational profiling, combined with inhibitors. Single-molecule fluorescence resonance energy transfer analysis of purified ribosomes represents a powerful complementary approach to determine the impact of specific nucleotide modifications on translational kinetics and ribosomal function, and cryo-electron microscopy may be used to visualize rRNA modifications at low A° resolution. These are some of the methods that are rapidly emerging as powerful tools for investigating ribosome specialization with exceptional resolution.
